# Electrophysiological signatures of temporal context in the bisection task

**DOI:** 10.1007/s00221-023-06670-1

**Published:** 2023-07-17

**Authors:** Cemre Baykan, Xiuna Zhu, Artyom Zinchenko, Hermann J. Müller, Zhuanghua Shi

**Affiliations:** General and Experimental Psychology, Department of Psychology, Ludwig-Maximilians-Universität München, Leopoldstr. 13, 80802 Munich, Germany

**Keywords:** Time perception, EEG, Temporal bisection, CNV, P2, LPCt

## Abstract

Despite having relatively accurate timing, subjective time can be influenced by various contexts, such as stimulus spacing and sample frequency. Several electroencephalographic (EEG) components have been associated with timing, including the contingent negative variation (CNV), offset P2, and late positive component of timing (LPCt). However, the specific role of these components in the contextual modulation of perceived time remains unclear. In this study, we conducted two temporal bisection experiments to investigate this issue. Participants had to judge whether a test duration was close to a short or long standard. Unbeknownst to them, we manipulated the stimulus spacing (Experiment 1) and sample frequency (Experiment 2) to create short and long contexts while maintaining consistent test ranges and standards across different sessions. The results revealed that the bisection threshold shifted towards the ensemble mean, and both CNV and LPCt were sensitive to context modulation. In the short context, the CNV exhibited an increased climbing rate compared to the long context, whereas the LPCt displayed reduced amplitude and latency. These findings suggest that the CNV represents an expectancy wave preceding a temporal decision process, while the LPCt reflects the decision-making process itself, with both components influenced by the temporal context.

## Introduction

Processing the vast amount of information that surrounds us can be challenging, as our sensory organs have limited processing capacity (Wolfe 1994), and more so, our memory and attentional resources (Cavanagh and Alvarez 2005). To overcome these limitations, our brain has developed ensemble perception (Whitney and Yamanashi Leib 2018), a mechanism that allows us to quickly grasp the essence of a scene by extracting statistical information, such as the mean and variance, from its features. For example, we can effortlessly estimate the average size of apples in a supermarket by simply glancing at them, without a need to analyze each individual apple in detail. Similar forms of ensemble perception are used to process basic features, including average motion, orientations, colors (Albrecht et al. 2012; Ariely 2001; de Gardelle and Summerfield 2011; Parkes et al. 2001; Piazza et al. 2013; Williams and Sekuler 1984), and sequential durations (Zhu et al. 2021). There are two types of ensemble representations: spatial (Whitney and Yamanashi Leib 2018) and temporal (Jones and McAuley 2005; Schweickert et al. 2014). Spatial ensemble representation involves representing a group of similar objects presented simultaneously, while temporal ensemble representation involves processing a sequence of stimuli over time.

Both types of ensemble statistics can influence judgments of individual items when they serve as context (Ariely 2001; Zhu et al. 2021). For instance, in a temporal bisection task, where participants judge whether a probe duration is closer to a fixed “short” or “long” anchor, it was previously believed that the judgment solely relies on the probe’s proximity to the anchors. However, studies have demonstrated that the spacing of the probe durations (Allan 2002; Penney and Cheng 2018; Wearden and Ferrara 1995) and the sample distribution (Zhu et al. 2021) can affect our judgments, shifting the transition point between “short” and “long” responses, known as the bisection point (BP). This bias, known as *the spacing effect*, occurs when there are uneven time intervals among the sampled durations (Wearden and Ferrara 1995). In addition, *the range effect* occurs when the spread of the sample set influences judgments of its individual durations (Droit-Volet and Wearden 2001; Penney et al. 2014; Wearden and Ferrara 1996). While our understanding of the behavioral effects of temporal contextual modulation has improved (Zhu et al. 2021), the neural mechanisms underlying these common temporal context effects are not yet fully elucidated, although recent research has provided some insights into this topic (Damsma et al. 2021; Wiener et al. 2018; Wiener and Thompson 2015).

Recent EEG studies have identified several event-related potentials (ERP) associated with time processing and contextual modulation (Lindbergh and Kieffaber 2013; Ng et al. 2011; Wiener and Thompson 2015). In the context of a bisection task, the contingent negative variation (CNV)—a negative polarity waveform typically observed over frontocentral brain regions—has been found to increase in negativity as the interval progresses and to level off when the duration exceeds the geometric mean of the short and long anchors (Ng et al. 2011; van Rijn et al. 2011; Wiener and Thompson 2015). In addition, subsequent post-interval positivity ERPs, which appear in the same electrode clusters as CNV and occur in the range between 200 and 600 ms after the stimulus offset, have been found to vary with duration judgments and temporal decisions (Damsma et al. 2021; Ofir and Landau 2022). An early ERP positivity component, known as P2, peaking around 200 ms after the stimulus, has been suggested to be linked with perceived duration length (Kononowicz and van Rijn 2014), although the exact relationship between P2 and the probe duration remains unclear (Kononowicz and van Rijn 2014; Lindbergh and Kieffaber 2013). Late positivity components, such as P3, P3b, or the late positive component related to timing (LPCt), measured approximately 300–600 ms after the stimulus, have been associated with temporal decision-making (Bannier et al. 2019; Lindbergh and Kieffaber 2013; Paul et al. 2011). For instance, Ofir and Landau (2022) revealed a negative correlation between the offset-evoked P3 and stimulus duration in a bisection task, with the amplitude decreasing as the stimulus duration increased. Using a drift–diffusion model, they predicted behavioral performance by assuming that the amplitude of the offset P3 reflects the proximity of temporal accumulation to the decision boundary (i.e., the bisection point). Similarly, LPCt has been found to vary with the probe duration, with larger positive amplitudes associated with shorter durations (Wiener and Thompson 2015). Given that LPCt or P3 is measured after the duration offset, higher peaks for the short compared to long intervals are interpreted as an indication that decisions for short intervals are more demanding, as the decision process remains active and unresolved at the offset of a short presentation (Lindbergh and Kieffaber 2013).

Early studies primarily focused on ERP components related to temporal memory, without considering contextual modulation (e.g., Macar et al. 1999). However, recent studies have shown that these ERP components are also sensitive to temporal contexts. Wiener and Thompson (2015) found that both the CNV and LPCt covaried with the duration presented in the preceding trial. Damsma et al. (2021) conducted a study in which participants were asked to reproduce intervals from two different but overlapping ranges (short and long). When the same interval was reproduced in the short-range session compared to the long-range session, higher amplitudes of CNV and offset P2 were observed. Furthermore, the amplitudes of CNV and offset P2 decreased as the preceding interval increased. By probing a bisection task separately for subsecond and supra-second ranges, Ofir and Landau (2022) found that the amplitude of offset P3 exhibited a similar pattern in both ranges, highlighting the nature of contextual modulation (Baykan and Shi 2022).

It should be noted that decisions in a bisection task can be made *during* stimulus presentation without waiting until the end, as it becomes clear whether a duration is short or long once the elapsed time passes the bisection point between the short and long anchors. But decisions in a reproduction task can only be made during the late reproduction phase, after the complete presentation of the duration. Consequently, ERP components associated with different timing tasks, such as bisection or reproduction, may reflect different temporal cognitive processes depending on the task employed (Gontier et al. 2009; Kononowicz and van Rijn 2014; van Rijn et al. 2011).

It is important to acknowledge that the aforementioned EEG studies primarily focused on neural activities during the probe itself. Although some studies have touched upon contextual modulation (e.g., Ofir and Landau 2022), the contexts examined often involved substantial differences, such as sampling from different duration ranges (e.g., subseconds vs. supra-seconds). None of these studies has explored *ensemble contexts* within the same duration range but with variations in stimulus spacing or sample frequencies. Consequently, neurophysiological mechanisms underlying such *ensemble contexts* remain poorly understood. To address this gap, we conducted two experiments using the bisection task and manipulated the sampled durations. In both experiments, the short anchor was set at 400 ms, and the long anchor at 1600 ms. Participants were required to judge whether a probe duration was closer to the short standard or the long standard. Unbeknownst to the participants, in Experiment 1, the sampled durations were positively skewed in one session and negatively skewed in the other, while in Experiment 2, one session included high sample frequencies of short durations and the other session included high frequencies of long durations. Building upon previous findings (Mento et al. 2013; Ng et al. 2011; Wiener and Thompson 2015), we hypothesized that the peak latencies of CNV would correlate with the internal decision criterion of the bisection task. Specifically, we expected earlier peak latencies in short contexts compared to long contexts, reaching a plateau after the ensemble mean duration. Consistent with the literature (Kononowicz and van Rijn 2014; Tarantino et al. 2010), which suggests that the amplitude of P2 is linked to the stimulus magnitude, we predicted that the amplitude of P2 would increase with the target interval and be more positive in short relative to long contexts. To distinguish the late positivity components (e.g., LPCt) from the P2 component, we introduced a 300 ms blank interval after the stimulus offset in Experiment 2 to examine the relationship between the LPCt amplitude and the target interval.

## Experiment 1

In Experiment 1, we manipulated the temporal context using the positively skewed (PS, more short durations) and negatively skewed (NS, more long durations) sample distributions, based on our previous work (Zhu et al. 2021). Behaviorally, we expected the same outcome as the previous study—intermediate durations would be more likely to be judged as “long” in the PS than in the NS context.

### Methods

#### Participants

20 participants with no hearing impairment took part in Experiment 1 in exchange for a monetary reward or course credit at LMU Munich. The sample size was calculated based on the effect size of a similar temporal bisection study (Zhu et al. 2021) with ${\eta }_{g}= .26$, and the assumption of α = 0.05 and power 1–β = 0.95, which required a sample size of 16 participants. To be safe for EEG analysis, we increased the sample size to 20. All participants provided written informed consent before their participation. One participant was excluded from the formal analysis because of excessive eye and body movement artifacts. Thus, the final sample included 19 participants (10 females, mean age 27.2 years, *SD* = 4.2 years), who were naive to the purpose of the study. The study was approved by the Ethics Board of the Department of Psychology at LMU Munich.

#### Stimuli and procedure

The auditory stimuli were generated using the PsychoProtAudio library and presented through loudspeakers (Logitech Z130) using the Psychtoolbox 3 (Kleiner et al., 2007). Instructions and feedback text were displayed on a CRT monitor.

**Fig. 1:**
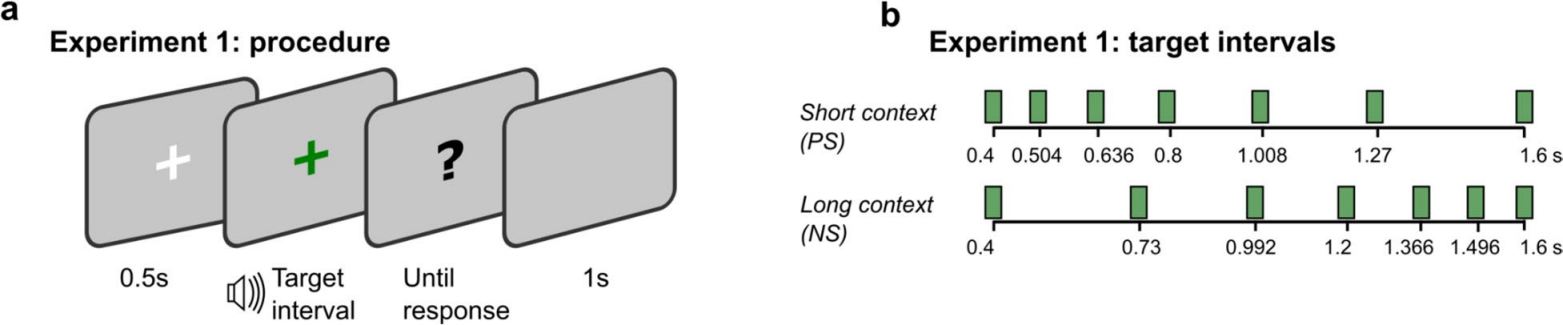
**a** Each trial started with fixation cross for 500 ms. It was followed by a target interval presentation. Right after the presentation, a question mark appeared, prompting participants to respond. The inter-trial interval was 1000 ms. **b** The target intervals used in Experiment 1. In the short-context session (PS), intervals were logarithmically spaced between 400 and 1600 ms, and the intervals were mirrored in the long-context session (NS). Each target interval was presented 48 times during the session

Participants sat in a sound-attenuated, moderately lit test room. Prior to the formal experiment, participants received a practice block consisting of 5 presentations of the short and long anchors (400 and 1600 ms) to familiarize themselves with the anchors. During the practice, participants made “short” or “long” judgments and received feedback on whether they were correct or incorrect. In the formal test, each trial started with a visual fixation and a brief beep (20 ms, 1000 Hz, 60 dB), followed by a 500 ms blank display, signaling the start of a new trial. A white-noise stimulus (60 dB) was then presented for a given duration chosen from the experimental stimulus sets (see below). Immediately after the sound presentation, a question mark appeared, prompting participants to respond by pressing the right or left arrow keys on the keyboard using two index fingers, indicating if the presented sound was close to the short or the long, respectively (Fig. 1a).

There were two sessions with each session of 336 trials (six blocks of 56 trials each). Two sessions had different duration sets: the positively skewed (PS) duration set consisted of [400, 504, 636, 800, 1008, 1270, 1600] ms, the negatively skewed (NS) duration set of [400, 730, 992, 1200, 1366, 1496, 1600] ms (Fig. 1b). The ensemble mean of the NS was 223 ms longer than the ensemble mean of the PS context. Each duration was randomly tested 48 times. The order of sessions was counterbalanced across participants (before the outlier exclusion).

#### EEG acquisition and analysis methods

Electrical brain activity was recorded from 64 scalp locations (actiCAP system; Brain Products, Munich, Germany) using the BrainVision Recorder software (Brain Products GmbH, Munich, Germany) and a BrainAmp amplifier (DC to 250 Hz) at the sampling rate of 1000 Hz. During the experiment, the impedances of all electrodes were kept below 10 kΩ. The electrode FCz was used as an online reference and EEG data were re-referenced to temporal-parietal electrodes offline (TP9 and TP10).

The EEG data were analyzed using BrainVisionAnalyzer 2.0 software, with a bandpass filter of 0.1 to 70 Hz. Artifacts caused by eye blinks, eye movements and muscle noises were removed using independent component analysis (ICA) and visual identification. Before segmentation, the continuous EEG data were inspected automatically using the raw data inspection procedure in the analyzer software and were bandpass-filtered from 0.1 to 30 Hz.

#### ERP components

All ERP components reported here were calculated for each participant, target interval, and temporal context. The onset-locked ERP data for CNV analyses were baselined to the average voltage 200 ms prior to the stimulus onset, using six clustered frontocentral electrodes FCz, FC1, FC2, C1, C2 and Cz (Kononowicz and van Rijn 2014; Ng et al. 2011). Given the negative ballistic deflation of the activities after post-onset P2, we examined the evolving velocity or climbing rate of the CNV negativity using linear regression within the time window from 250 (after P2) to 650 ms (the start of CNV) and obtained slopes for individual participants in each condition. We extracted the CNV peak latencies as the minimum (most negative) amplitude from stimuli onsets to the longest duration offset (i.e., 1600 ms) for each target interval in each context. We then calculated the CNV peak amplitudes as the averaged amplitude of 10 ms surrounding the CNV peak (5 ms preceding and 5 ms following the peak). We defined the mean CNV amplitude of each target interval as the average waveform in the interval starting from the late negativity onset (250 ms after the onset) and having a length of the stimulus duration (Kruijne et al. 2021). The stimulus offset P2s were calculated using the same frontocentral electrodes as used for the CNV analysis (Damsma et al. 2021). The offset-locked ERP data for P2 analyses were baselined to the 100 ms time window surrounding the stimulus offset (50 ms preceding and 50 ms following the offset) (Kononowicz and van Rijn 2014). We extracted the P2 peak latencies as the maximum (most positive) amplitude within the 0–500 ms duration offset window. We defined the mean P2 amplitude of each target interval as the average waveform between 140 and 300 ms after the stimulus offset (Kononowicz and van Rijn 2014).

#### Data analysis

Psychometric functions were estimated using the logistic function with the Quickpsy package in R (Linares and López-Moliner 2016), the point of subjective equality (PSE) was then calculated at the threshold of 50%, and the just noticeable difference (JND) as the difference between the thresholds at 50 and 75%. Mean PSEs and JNDs were compared using paired *t-*tests. For analysis of the EEG components, we applied a linear mixed model, which can accommodate the covariant factor (duration) in addition to the fixed effects addressed by ANOVA. Mixed models are robust to violations of sphericity and do not inflate Type I errors (Singmann and Kellen 2019). The *p*-values reported for mixed models were calculated using the Kenward–Roger approximation.

### Results

#### Behavioral results

**Fig. 2:**
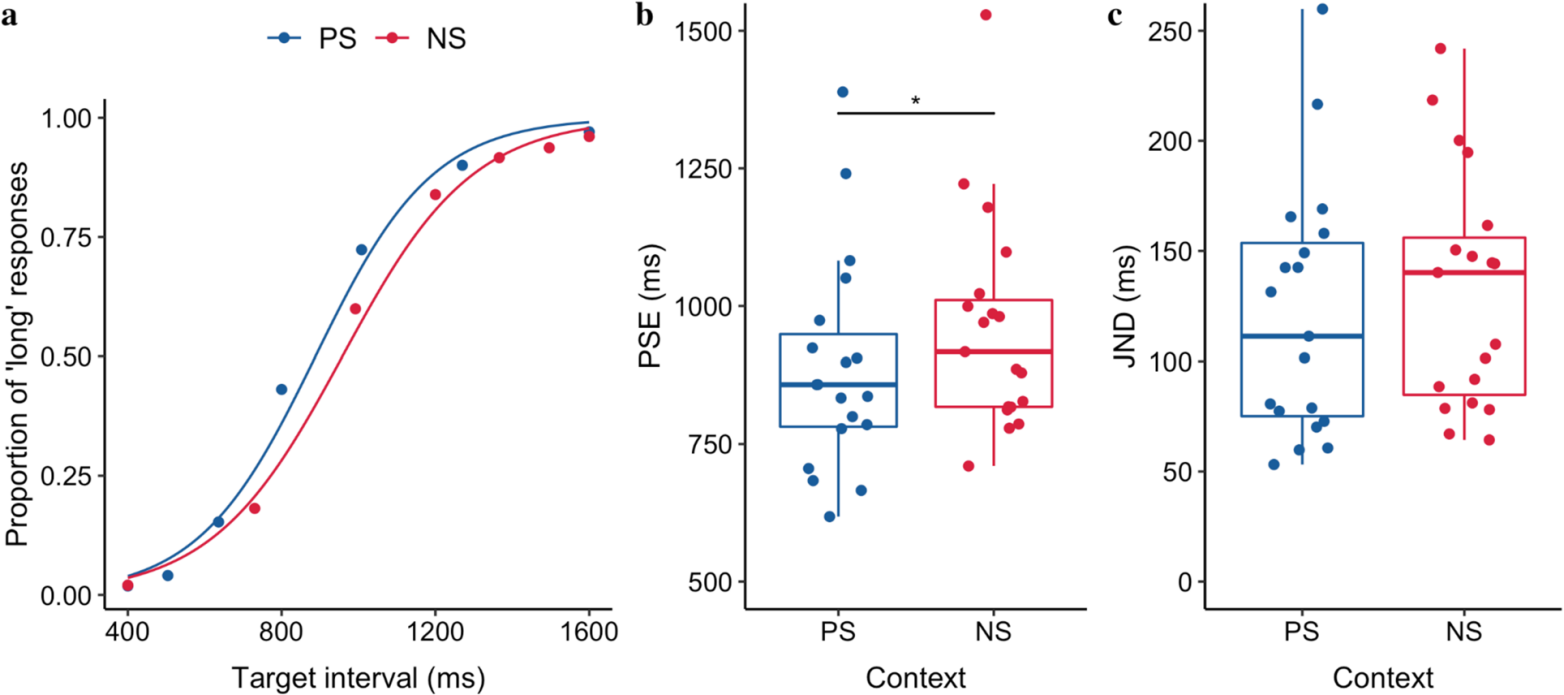
**a** The averaged proportion of ‘long’ responses (scatter dots) and the fitted psychometric curves over 19 participants, for the positively (PS) and negatively skewed (NS), stimulus-spacing conditions. **b** Boxplots of the points of subjective equality (PSEs) of the duration judgments for the PS and NS sessions (* *p < *.05). The dots depict individual PSEs. The lower and upper tips of the vertical lines correspond to the minimum and maximum values, the box the interquartile range (between 25 and 75%), and the horizontal line the median. **c** Boxplots of JND of the duration judgments for the PS and NS sessions. The dots depict individual JNDs

Figure 2 illustrates the averaged psychometric functions, mean PSEs and JNDs. The mean PSE (± standard error, *SE*) for the short-context PS session was significantly shorter (888.7 ± 45.9 ms) than the long-context NS session (958.9 ± 46 ms), *t*(18) = − 2.63, *p* = 0.017, 95% CI = [− 126.25 to − 14.02] ms, *BF* = 3.04 (see also Table 1). In other words, the same duration (e.g., 1 s) was perceived longer in the short relative to the long context. The sensitivities of the bisection, measured by JNDs, were comparable between the two sessions, *t*(18) = − 1.56, *p* = 0.14, 95% CI = [− 24.89 to 3.65] ms, *BF* = 0.74, indicating the spacing of the target intervals did not change the discrimination sensitivity. Thus, the behavioral results are in line with the previous findings (Zhu et al. 2021).

**Table 1:** Mean ERPs and behavioral PSEs and JNDs

Experiment	Context	CNV				Behavioral	
		Climbing rate (μV/s)	Peak latency (ms)	Peak amplitude (μV)	Mean amplitude (μV)	PSE (ms)	JND (ms)
1: Spacing	Short (PS)	**− 23 ± 1.9**	**773.2 ± 34.2**	**− **6.1 ± 0.6	**− **1.47 ± 0.6	**888.7 ± 46**	121.1 ± 13.4
	Long (NS)	**− 20 ± 1.6**	**875.9 ± 49.3**	**− **5.6 ± 0.6	**− **1.17 ± 0.5	**958.9 ± 46**	131.7 ± 12.7
2: Frequency	Short (DF)	**− 19 ± 1.5**	942.8 ± 40.9	**− 5.4 ± 0.5**	**− **0.78 ± 0.4	**749.3 ± 40**	**96.3 ± 6.7**
	Long (AF)	**− 17 ± 1.5**	876.6 ± 67.7	**− 4.5 ± 0.5**	**− **0.27 ± 0.4	**951.2 ± 50**	**121.1 ± 12.1**

The mean values (± standard errors of the mean) across participants. The data are grouped by temporal contexts in both Experiments 1 and 2. Bold values indicate a significant difference between the two contexts.

#### Electrophysiological results

##### Contingent negative variation (CNV)

**Fig. 3:**
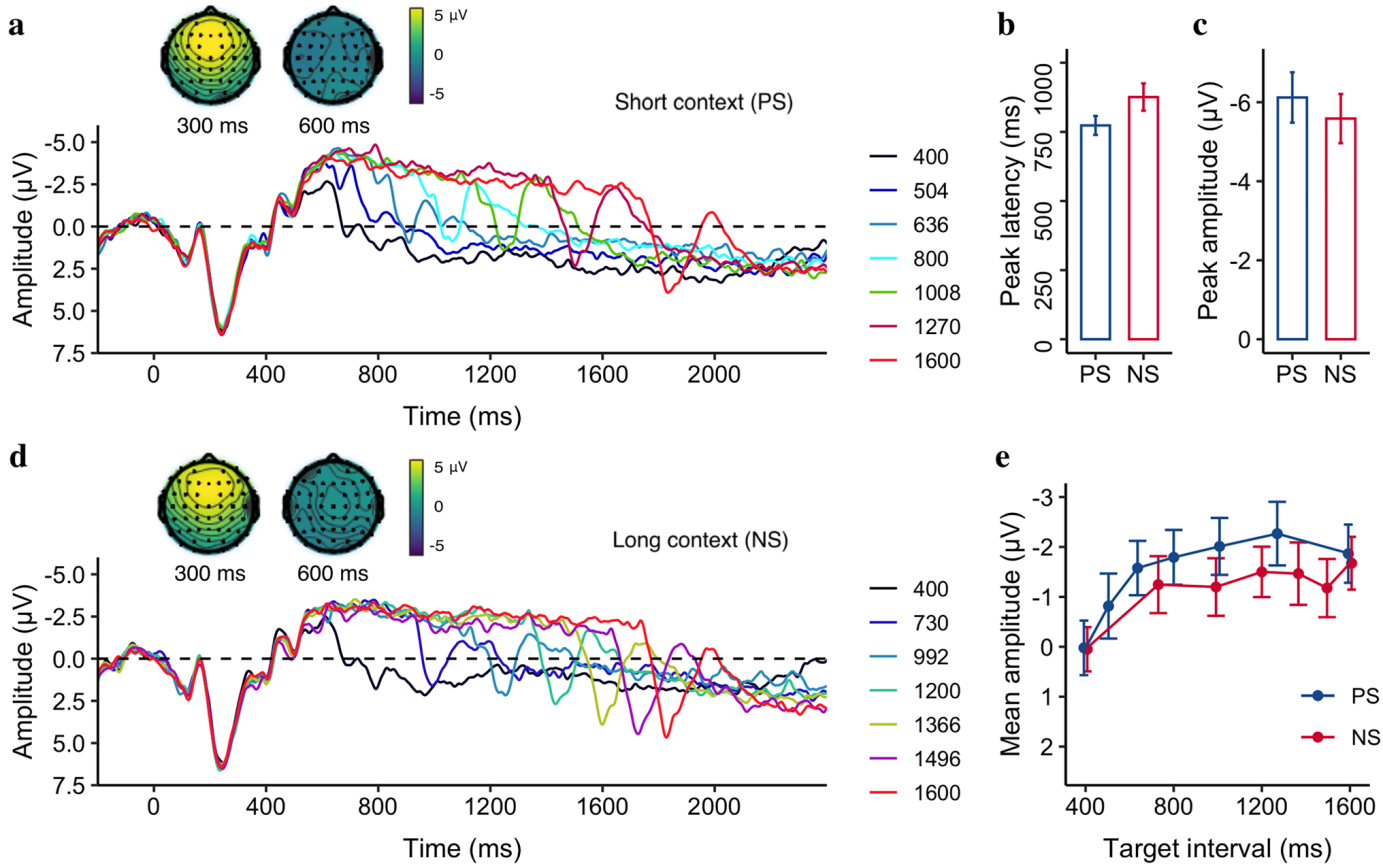
**a** Grand average of the ERP waveforms over the medial frontal electrodes (FCz, FC1, FC2, C1, C2, and Cz), separated for different target intervals, separated for **a** the short (PS) and **d** the long (NS) contexts. ERP topographies of the onset P2 and CNV at 300 and 600 ms, respectively, taken from the longest interval of both contexts. The mean CNV peak latency **b** and amplitude **c** of the target intervals, separated for the PS and NS conditions. **e** The mean CNV amplitude as a function of the target interval, separated for the PS and NS conditions. Each error bar represents the standard error of its mean

Figure 3 illustrates the CNV activities in the short PS (a) and long NS contexts (d), showing the negativity changes over time for different target intervals. To characterize the CNV component, we looked into its climbing formation rate, peak latency, peak amplitude, and mean amplitude. The mean values and their associated standard errors are listed in Table 1.

We found the rate was significantly negative for both the PS context (− 23 ± 1.9 μV/s, 95% CI = [− 27 to − 19] μV/s, *t* (18) = − 12.24, *p < *0.001) and the NS context (− 20 ± 1.6 μV/s, 95% CI = [− 24 to − 17] μV/s, *t* (18) = − 13.12, *p < *0.001), but significantly smaller in the PS compared to the NS, *t*(18) = − 3.01, *p* = 0.01, 95% CI = [− 4.08 to − 0.72] μV/s, *BF* = 5.53. Moreover, the CNV peaked significantly earlier for the short-context PS relative to the long-context NS (773.2 ± 34.2 ms vs. 875.9 ± 49.3 ms, *t*(18) = -2.23, *p* = 0.04, 95% CI = [− 199.2 to − 6.1] ms, *BF* = 2.17) (see Fig. 3b), but only numerically higher in amplitude for the PS relative to the NS (− 6.1 ± 0.6 μV vs. − 5.6 ± 0.6 μV, *t*(18) = − 1.9, *p* = 0.07, 95% CI = [− 1.12 to 0.06] μV, *BF* = 1.12) (see Fig. 3c).

As research has shown the mean amplitude of CNV to be correlated with the sample duration (Macar et al. 1999; Pfeuty et al. 2003, 2005), we estimated the mean amplitude separately for individual durations, as depicted in Fig. 3e. The averaged CNV mean amplitudes (± *SE*) were − 1.47 ± 0.6 μV and − 1.17 ± 0.5 μV for the PS and NS context, respectively. We applied a linear mixed model to the mean CNV amplitude, with the Context as the fixed effect and Duration a covariant effect. The mixed model showed that the mean negativity increased by 1.23 μV, per second of Duration (*b* = − 1.23, *CI* = [− 1.72, − 0.73], *p < *0.001). However, there was no significant difference between the short and long contexts (*p* = 0.53) and no significant interaction between the Duration and Context (*p* = 0.31).

##### Offset P2

**Fig. 4:**
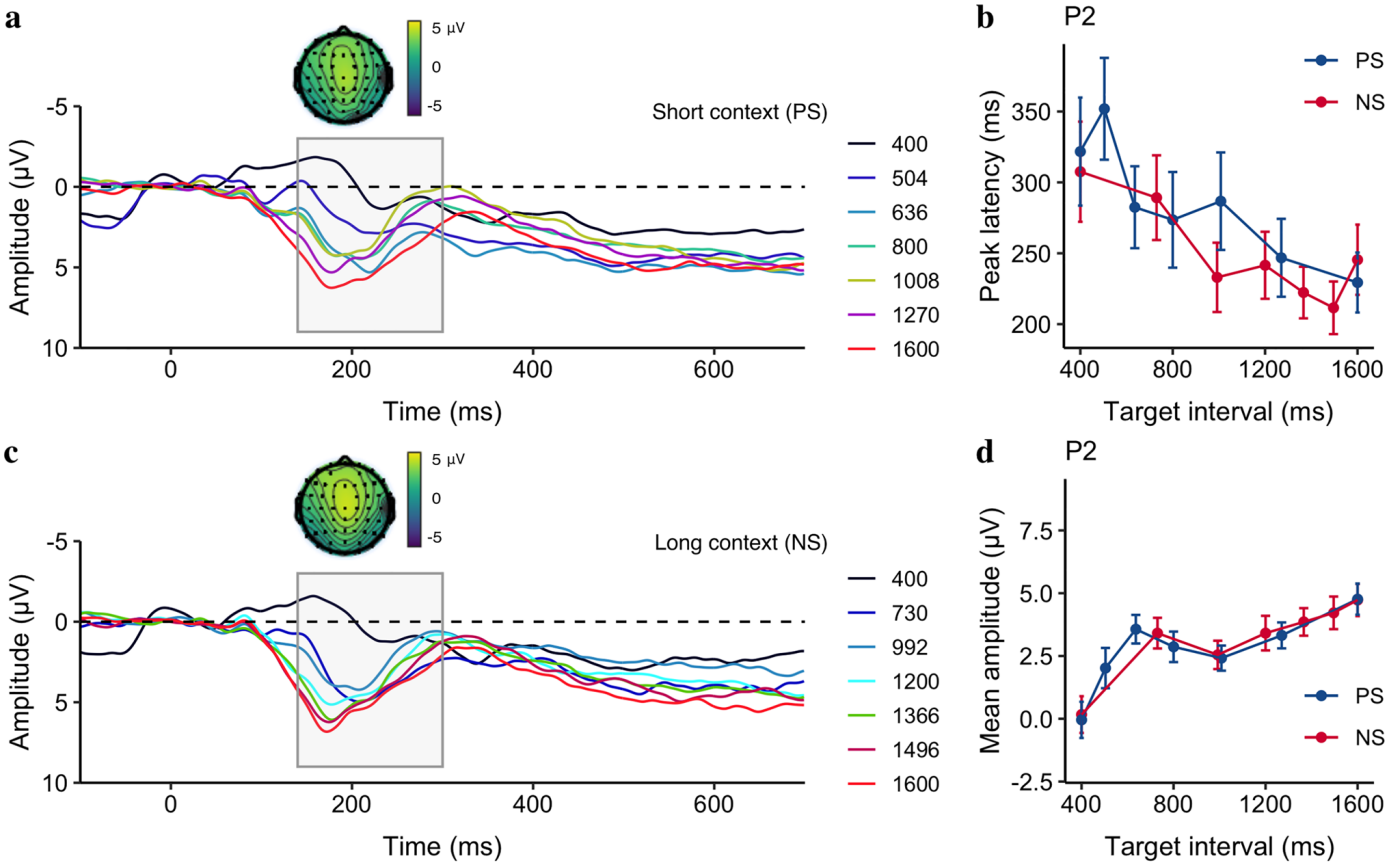
Grand average of the ERP waveforms over the medial frontal electrodes (FCz, FC1, FC2, C1, C2, and Cz), separated for different target intervals, separated for the PS **a** and the NS conditions **c**. ERP topographies of the offset P2 at 200 ms after the stimulus offset taken from the longest interval of both contexts. **b** Mean P2 peak latency and **d** mean P2 amplitude as a function of the target interval, separated for the PS (blue) and NS (red) contexts. Error bars represent the corresponding standard errors

Figure 4a and 4c depict the ERP waveforms over the medial frontal electrodes relative to the offset of the stimuli for the short and long contexts, showing a positive peak around 200 ms after the stimuli offset that correlates with the target interval. Figure 4b and 4d show the peak latency and mean amplitude of the offset P2 as a linear trend of the target interval, separated for the PS and NS contexts: The latency decreases, but the amplitude increases as the target interval increases, while there was no significant difference between the two contexts. The averaged offset P2 peak latencies (± *SE*) were 284.7 ± 19.7 ms and 250.1 ± 15.9 ms for the PS and NS context, respectively. A linear mixed model with the Context as the fixed effect and Duration as a covariant effect was applied to the offset P2 peak latency, which revealed that the peak latency decreased by 78 ms/s of Duration (*CI* = [− 144.92, − 10.38], *p* = 0.026). But the peak latency showed no significant difference between the PS and NS Context (*p* = 0.28). Moreover, there was no significant interaction between Duration and Context (*p* = 0.58). The averaged offset P2 peak amplitudes (± *SE*) were 7.35 ± 0.86 μV and 7.14 ± 0.87 μV for the PS and NS context, respectively. Similar linear mixed model applied to the Offset P2 mean amplitudes (Fig. 4d) revealed a significant main effect of Duration (*b* = 2.90, *CI* = [1.98, 3.81], *p < *0.001). Again, there was no significant Context (*p* = 0.28) effect and no interaction between the Duration and Context (*p* = 0.41). The findings indicate that while the offset P2 was responsive to the target interval, it was insensitive to variation of ensemble contexts.

### Discussion

Experiment 1 replicated previous research (Wearden and Ferrara 1995; Zhu et al. 2021), confirming that temporal bisection is subjective to the target spacing. Intervals in the short context (PS), relative to the long context (NS), tended to be judged longer, indicating that participants not merely compared the probe duration to the short or long standards (which were the same in both contexts), but also took into account the spacing of the sample durations.

Experiment 1 revealed that the mean amplitude of CNV was linked to the target duration. As the duration increased, so did the mean amplitude. Figure 3e also shows that the mean amplitude leveled off at middle durations (800 to 1200 ms), which is in line with previous research (Macar and Vidal 2003; Ng et al. 2011) that found that CNV plateaued at the geometric mean of the short and long intervals in the bisection task. Moreover, the current results showed that the CNV climbing up rate was steeper in the short context compared to the long context. These findings support that CNV stands for temporal expectation (Amit et al. 2019; Praamstra et al. 2006). Some researchers have suggested that the CNV amplitude is subjective to the context. For example, adapting to shorter durations would lead to an increase in the amplitude of CNV, while adapting to longer durations would decrease the amplitude of the CNV (Li et al. 2017). Here, we found the mean (or peak) amplitude of the CNV was higher for the short context (PS) than for the long context (NS), but the difference did not reach statistical significance. On the other hand, we did find that the latency of the CNV was earlier for the short context relative to the long context, which aligns with previous research showing a faster development of the CNV activity for short than long target durations (Pfeuty et al. 2005). However, as Kononowicz and Penney (2016) have suggested, timing is not the only factor contributing to the CNV. More complex processes, such as preparation for an upcoming event, could play a role. Therefore, in some cases, the CNV may not truly reflect the temporal interval itself, as revealed in a previous study that CNV-like negativity simply disappears for intervals longer than 4 s (Elbert et al. 1991).

In addition, the latency of the offset P2, a common component associated with temporal accumulation as reported previously (Kononowicz and van Rijn 2014; Tarantino et al. 2010), had a negative correlation with the target interval, which is consistent with previous findings (Kononowicz and van Rijn 2014). This can be explained by the predictive coding account (Friston and Kiebel 2009; Kononowicz and van Rijn 2014; Rao and Ballard 1999), because short intervals that stopped before the decision threshold (i.e., the bisection point) led to larger ‘prediction errors’ than long intervals, resulting in early P2 latencies. This is also in line with the previous studies showing that short durations lead to longer reaction times (e.g., Bannier et al. 2019). However, the offset P2 was not affected by the spacing modulation, which is in contrast to previous reports indicating that offset signals such as LPCt, peaking at around 300 ms post-offset (later than P2), can be influenced by the task difficulty (Paul et al. 2011), the prior trial duration (Wiener and Thompson 2015), or the sample set (Ofir and Landau 2022).

It is worth noting that in previous studies using bisection or duration comparison tasks, the durations used were typically longer than 800 ms (e.g., Ng et al. 2011). This allowed sufficient time for the CNV component to peak around 600 to 800 ms (see examples in Fig. 3). In the present study, we used two short intervals (400 ms, 504 ms) that were shorter than 600 ms. This caused the immature CNV to stop earlier in preparation for action, resulting in some distortion of the offset P2 component (as seen in Fig. 4). Consequently, comparing the P2 component across durations, particularly with the short durations, became less ideal. To address this issue and to separate the decision-making process from temporal encoding in a bisection task, we introduced a 300 ms gap before prompting a decision in Experiment 2. In addition, to generalize the contextual modulation, we varied sample frequency instead of stimulus spacing.

## Experiment 2

### Methods

#### Participants

20 participants with no hearing impairment took part in Experiment 2 in return for a monetary incentive or course credit at LMU Munich. The sample size was the same as in Experiment 1. All participants were naive to the purpose of the study and gave written informed consent before the formal experiment. The study was approved by the Ethics Board of the Department of Psychology at LMU Munich.

Because of the excessive eye or body movement artifacts during EEG recording, three participants were excluded from further analyses. Thus, the results of 17 participants (6 females, mean age 27.3 years, *SD* = 3.5 years) were reported here.

#### Stimuli and procedure

**Fig. 5:**
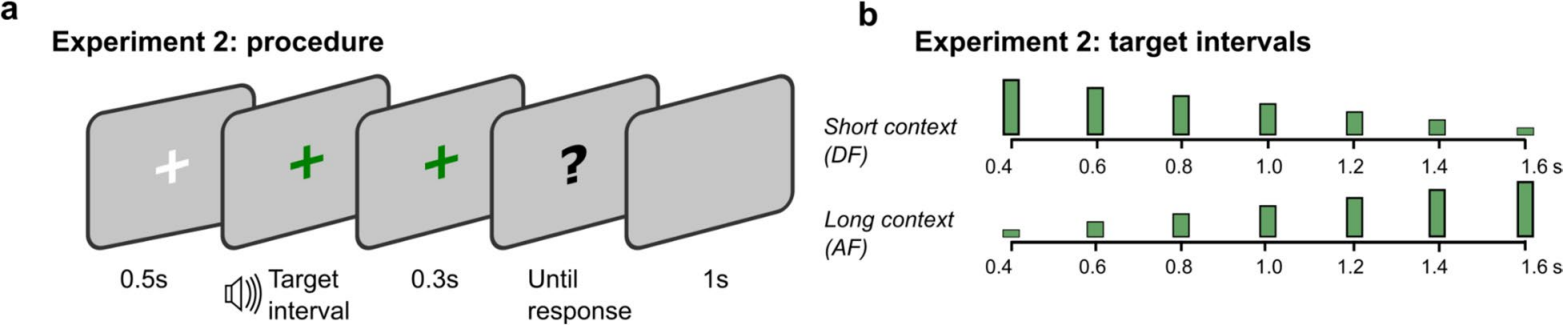
**a** Each trial started with a fixation cross for 500 ms, followed by a target interval presentation. 300 ms after the presentation, a question mark was presented, prompting participants to respond. The inter-trial interval was 1000 ms. **b** Target intervals used in Experiment 2. In the short-context session (DF), equally spaced intervals between 400 and 1600 ms were presented 84, 72, 60, 48, 36, 24, and 12 times during the session, whereas the presentation frequencies were mirrored in the long-context session (AF)

The experimental setup was the same as in Experiment 1, with the following two exceptions: first, a 300-ms blank was inserted between the stimulus offset and the question mark (prompting for a response), providing a decision time buffer for short durations (Fig. 5a); second, two sessions had the same equal-spaced duration set of [400, 600, 800, 1000, 1200, 1400, 1600] ms, but sampled with different frequencies (Fig. 5b). In one session, the above durations were tested [12, 24, 36, 48, 60, 72, 84] times, respectively. We referred to this session as the ascending frequency (AF) session. In the other, descending frequency (DF) session, the same durations were tested [84, 72, 60, 48, 36, 24, 12] times, respectively. Within each session, the durations were randomly selected with the respective frequency. The order of sessions was counterbalanced among participants (before the outlier exclusion).

#### ERP components

Similar to Experiment 1, we examined the CNV activity by measuring its climbing rates, latencies and amplitudes. The LPCt components were estimated on the same frontocentral electrodes as the CNV analysis (Damsma et al. 2021), but baselined relative to the 100 ms time window surrounding the onset of the question mark (50 ms preceding and following the question mark) (Kononowicz and van Rijn 2014). We extracted the LPCt peak latencies as the maximum (most positive) amplitude within the 500 ms window starting from the question mark. We then calculated the LPCt peak amplitudes as the averaged amplitude within 10 ms surrounding the LPCt peak (5 ms preceding and 5 ms following the peak). We defined the LPCt mean amplitudes as the averaged waveform between 300 and 500 ms after the stimulus offset (Bueno and Cravo 2021; Ofir and Landau 2022).

### Results

#### Behavioral results

**Fig. 6:**
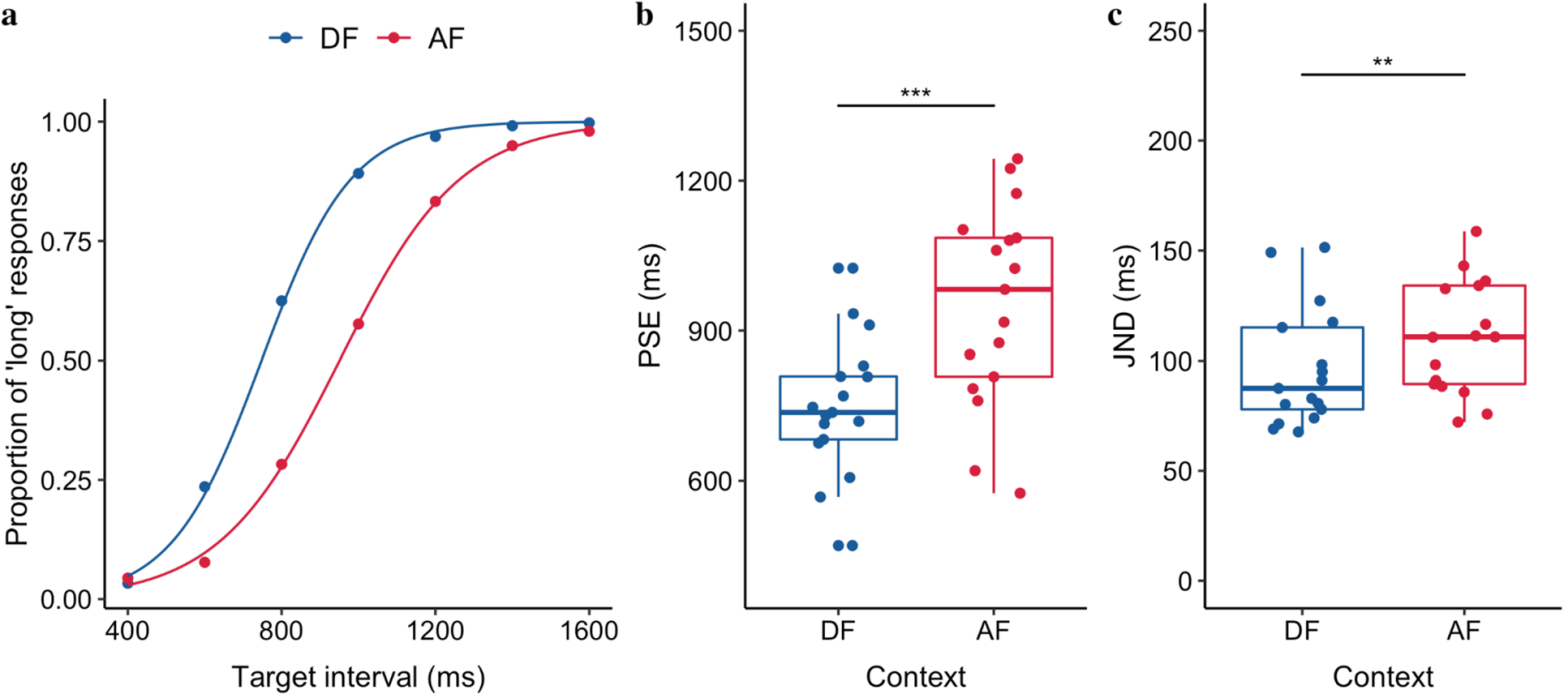
**a** Bisection functions (proportions of “long” responses plotted against the target durations, and fitted psychometric curves) averaged across 17 participants for the 2 distributions, descending (DF) and ascending frequency (AF). **b** Boxplots of PSE of the duration judgments for the DF and AF sessions (*** *p < *.001). The dots depict individual PSEs estimated from individual participants. The lower and upper tips of the vertical lines correspond to the minimum and maximum values, the box represents the interquartile range (between 25 and 75%), and the horizontal line represents the median. **c** Boxplots of JND of the duration judgments for the DF and AF sessions (** *p < *.01). The dots depict individual JNDs of individual participants

Figure 6a illustrates the averaged proportion of long responses and corresponding estimated psychometric functions. The mean PSE (± *SE*) was 749.3 ± 33.86 ms for the DF session, significantly shorter than for the AF session (951.2 ± 49.91 ms), *t*(16) = − 5.26, *p < *0.001, 95% CI = [− 283.20 to − 120.61] ms, *BF* > 100, indicating the durations in the DF session were perceived longer than the same durations in the AF session. This finding is consistent with the previous study (Zhu et al. 2021). Moreover, the mean JND (± *SE*) was 96.3 ± 6.69 ms for the DF session, significantly smaller than for the AF session (119.8 ± 12.09 ms), *t*(16) = − 2.97, *p < *0.01, 95% CI = [− 40.26 to − 6.72] ms, *BF* = 5.22, showing that the sensitivity of the bisection was higher in the DF compared to the AF session.

#### Electrophysiological results

##### The CNV

**Fig. 7:**
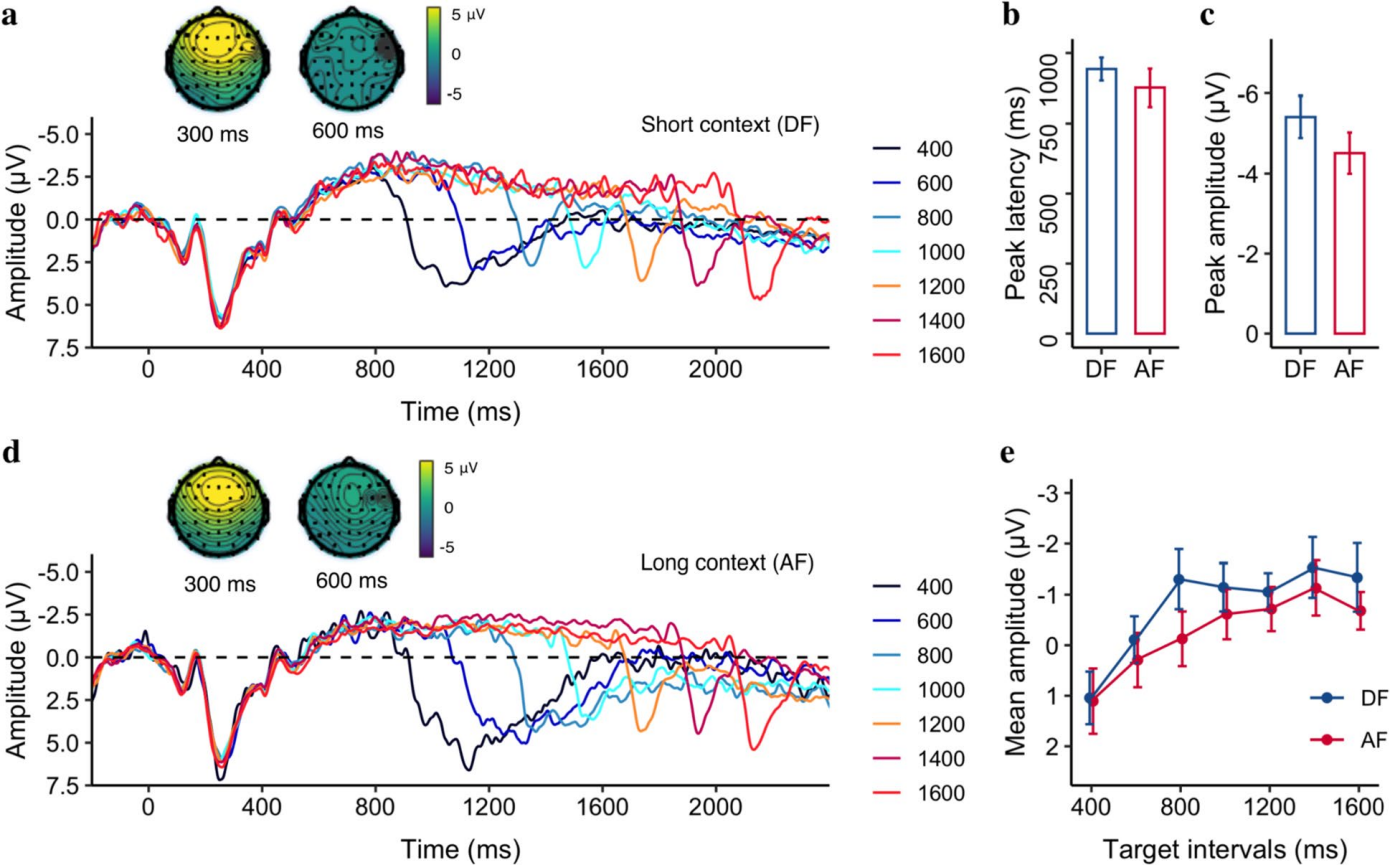
Grand average of the ERP waveforms over the medial frontal electrodes (FCz, FC1, FC2, C1, C2, and Cz) relative to the onset of stimuli, for **a** the short (DF) and **d** the long (AF) contexts. ERP topographies of the onset P2 and CNV at 300 and 600 ms, respectively, taken from the longest interval of both contexts. The mean CNV peak latency **b** and amplitude **c** of the target intervals, for the DF and AF conditions. **e** The mean CNV amplitude as a function of the target interval, for the DF and AF conditions. Error bars represent the standard error of its mean

Figure 7 illustrates the CNV activities both in the short DF (a) and long AF (d) contexts, showing that the negativity changes over time for different target intervals. Just like in Experiment 1, to characterize the CNV component, we looked into its climbing rate, peak latency, peak amplitude, and mean amplitude. The mean values are listed in Table 1.

We found that the rate was significantly negative for both the DF context [− 19 ± 1.5 μV/s, 95% CI = [− 22, − 15] μV/s, *t* (16) = − 12.57, *p < *0.001] and the AF context [− 17 ± 1.5 μV/s, 95% CI = [− 20 to − 14] μV/s, *t* (16) = − 11.37, *p < *0.001], but significantly smaller in the DF compared to AF context, *t*(16) = − 2.4, *p* = 0.03, 95% CI = [− 2.99 to − 0.18] μV/s, *BF* = 2.14. Moreover, the CNV peak amplitudes were significantly higher (− 5.4 ± 0.5 μV vs. − 4.5 ± 0.5 μV) for the short context (DF) relative to the long context (AF), *t*(16) = − 2.43, *p* = 0.03, 95% CI = [− 1.69 to − 0.11] μV, *BF* = 2.22), but with a comparable latency (942.8 ± 40.9 ms vs. 876.6 ± 67.7 ms, *t*(16) = 0.93, *p* = 0.37, 95% CI = [− 85.9 to 218.3] ms, *BF* = 0.49).

The mean amplitudes of CNV (± *SE*) were − 0.78 ± 0.42 μV and − 0.27 ± 0.44 μV for the DF and AF context, respectively. We applied a linear mixed model to the mean CNV amplitude, with the Context as the fixed effect and Duration as a covariant, which showed that the mean negativity amplitude increased by 1.66 μV for each second increase in Duration (*b* = − 1.66, *CI* = [− 2.35, − 0.96], *p < *0.001), demonstrating again that the CNV amplitude is correlated to the target interval. However, there was no significant Context (*p* = 0.47) effect or interaction between the Duration and Context (*p* = 0.71).

Last, to examine the correlation between the EEG responses and the behavioral data, the differences in the mean amplitudes of CNV and the PSEs between the long (AF) and short (DF) context were computed across all participants. A Pearson’s correlation test showed numerical positive correlation, *r*(15) = 0.32, but failed to reach any significance, *p* = 0.22, *BF* = 0.61.

##### Late positive component of timing (LPCt)

**Fig. 8:**
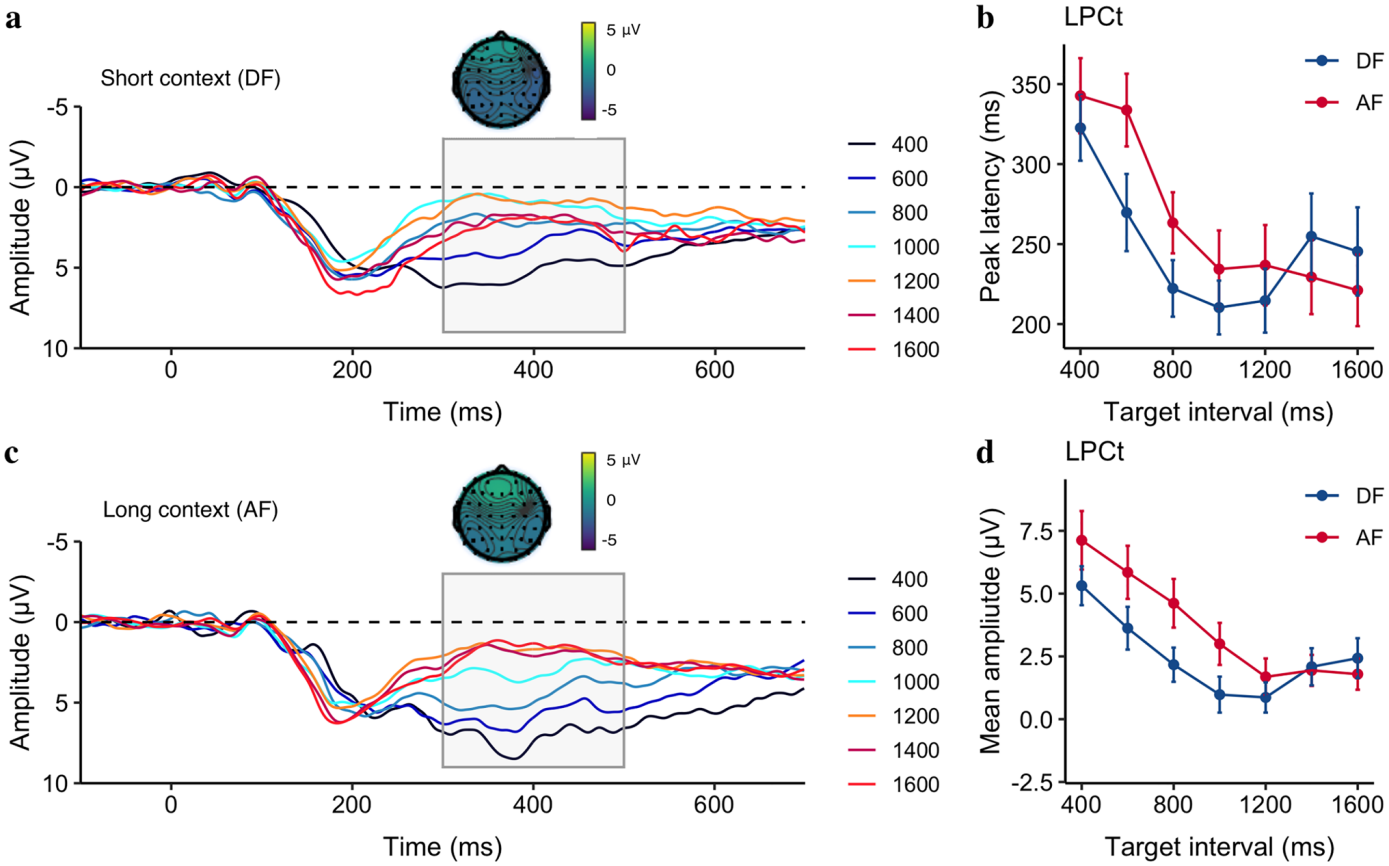
Grand average of the ERP waveforms over the medial frontal electrodes (FCz, FC1, FC2, C1, C2, and Cz) relative to the stimulus offset in the DF **a** and the AF conditions **c**. ERP topographies of the LPCt at 400 ms after the stimulus offset taken from the longest interval of both contexts. **b** Mean LPCt peak latency and **d** mean LPCt amplitude as a function of the target interval, separated for the DF (blue) and AF (red) contexts. Error bars represent the standard error of the correspondent mean

Next, we looked into the offset positivity components, such as P2 and LPCt, in the window of [0, 500] ms. Unlike Experiment 1, we failed to find any significant difference in the P2 component (the mean amplitudes were 4.2 ± 0.5 and 4.4 ± 0.6 for the DF and AF, respectively, *p* = 0.28. There was no significant difference among different target intervals, *p* = 0.99), but as seen in Fig. 8, there were visible differences in the late time window. Thus, we focused on the analysis of the LPCt component. The averaged LPCt peak latencies (± standard error, *SE*) were 248.6 ± 14.03 ms and 265.9 ± 11.87 ms for the DF and AF context, respectively (Fig. 8b). Same as in CNV analysis, we applied a linear mixed model to the LPCt peak latency, with the Context as the fixed effect and Duration as a covariant effect. The mixed model showed significant effects of Context (*b* = 38 ms, *CI* = [13, 63], *p* = 0.003), Duration (*b* = − 77 ms/s, *CI* = [− 133, − 22], *p* = 0.009) and the Duration × Context interaction (*b* = -30, *CI* = [− 53, − 6], *p* = 0.013). The LPCt peaked earlier for the short DF than the long AF context, and the latency decreased as the duration increased (see Fig. 8b).

For better comparison with the literature (Bueno and Cravo 2021; Ofir and Landau 2022), we extracted the mean LPCt amplitude from the time window of [300, 500] ms. The averaged LPCt mean amplitudes (± *SE*) were 2.49 ± 0.6 μV and 3.71 ± 0.7 μV for the DF and AF context, respectively. A similar linear mixed model on the LPCt mean amplitude (see Fig. 8d) revealed similar results: significant effects of Duration (*b* = − 3.55, *CI* = [− 5.13, − 1.97], *p < *0.001), Context (*b* = 1.83, *CI* = [1.09, 2.58], *p < *0.001) and the Duration × Context interaction (*b* = − 1.22, *CI* = [− 1.92, − 0.53], *p < *0.001). The mean amplitude was larger for the long AF than for the short DF context. As seen in Fig. 8d, the interaction was caused by different amplitudes for the short durations but plateaued at a similar level for the long durations.

To examine the correlation between the EEG responses and the behavioral data, the differences in the LPCt mean amplitudes and the JNDs between the long (AF) and short (DF) context were computed across all participants. A Pearson’s correlation test failed to reveal any significant correlation, *r*(15) = 0.01, *p* = 0.97, *BF* = 0.30.

#### Cross-experiment comparisons

**Fig. 9:**
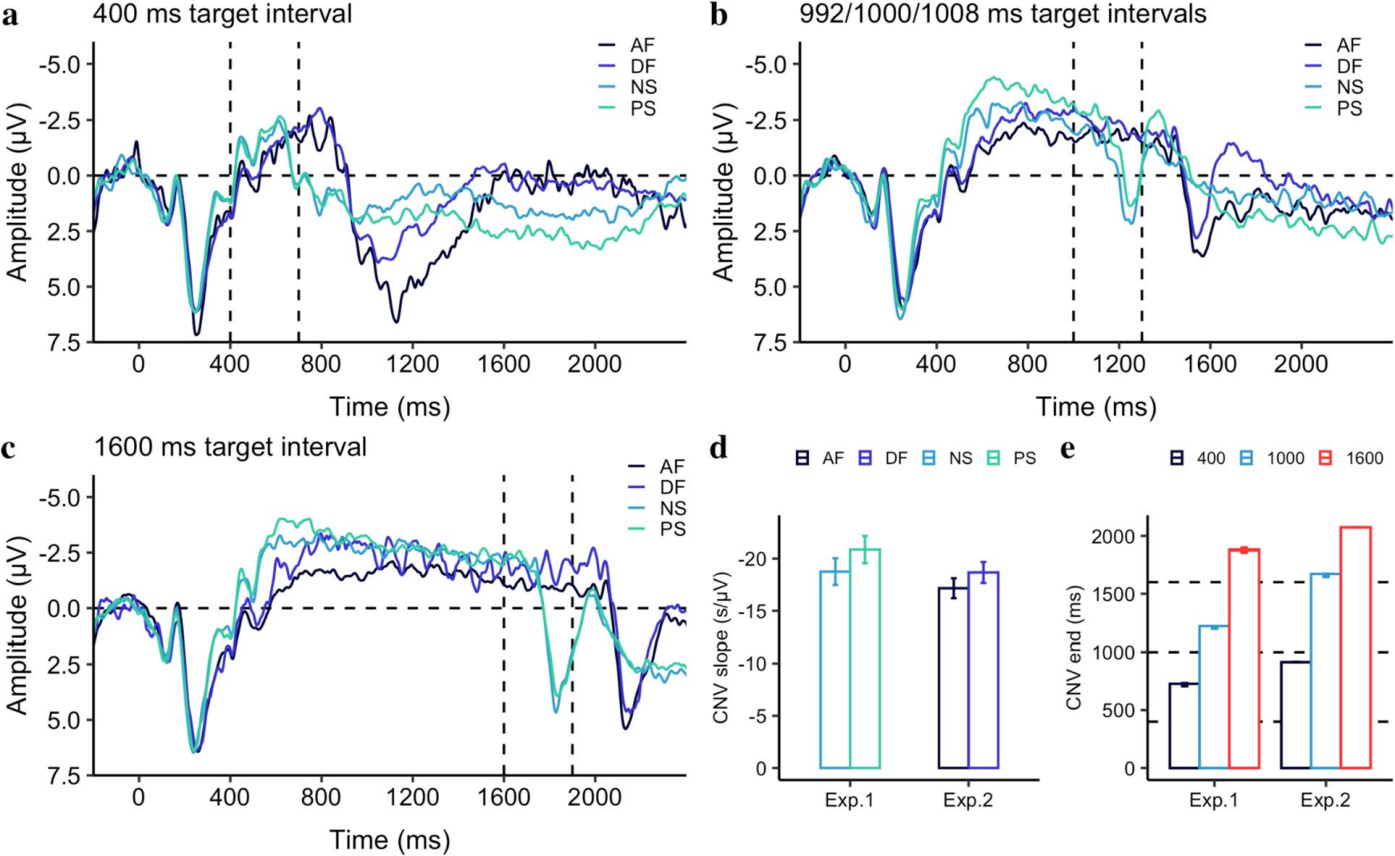
The grand average of the ERP waveforms over the medial frontal electrodes (FCz, FC1, FC2, C1, C2, and Cz) relative to the stimulus onset are depicted for the shortest **a**, intermediate **b,** and the longest **c** target intervals for the temporal contexts used in Experiment 1 and 2. Light blue and light green lines depict the long (NS) and short (PS) contexts of Experiment 1. Black and dark blue lines depict the long (AF) and short (DF) contexts of Experiment 2. The first vertical dashed line marks the offset cue (the question mark presentation) in Experiment 1, while the second vertical dashed line marks the offset cue in Experiment 2. **d** The CNV slope, measured in the interval from 250 to 650 ms after stimulus onset. Error bars show the standard error of the mean. **e** The CNV end, measured by the crossing point of the negativity waveform from negative to positive. Black and red colors depict the shortest and longest intervals (400 and 1600 ms) in both experiments, while the blue color depicts the intermediate durations of 992, 1000, and 1008 ms used in the experiments. For ease of visualization, they were depicted by the same color and label (1000 ms). Error bars show the standard error of the mean

To gain a better understanding of the temporal encoding process reflected in the CNV and the decision-making process reflected in the offset P2 and LPCt, we further compared the results of our two experiments for short (400 ms), intermediate (around 1000 ms), and long (1600 ms) durations (as shown in Fig. 9a, b, and c). Visual inspection shows that the CNV peaked earlier in Experiment 1 compared to Experiment 2. More interestingly, even when the duration was the same, the offset late positivity was delayed by about 300 ms, suggesting that the late positive component is not solely dependent on the offset of the duration, but also on the onset of the response (the onset of the question mark that prompts for response). Moreover, the late positivity component did not fully emerge for the short duration (400 ms) in Experiment 1, largely owing to the disruption of the ongoing CNV with immediate prompting for a response.

The mean slope of the CNV, measured within the interval from 250 to 650 ms, collapsed for these three intervals, was − 22 ± 1.1 μV/s for the short PS and − 19 ± 1.0 μV/s for the long NS in Experiment 1, while in Experiment 2, the mean slope was − 19 ± 1.0 μV/s for the short DF and − 17 ± 1.0 μV/s for the long AF. A linear mixed model was used to analyze the CNV slopes, with Experiment as the fixed effect and Context as the covariant effect. The analysis showed that there was a significant effect of Context (*b* = 1.14, *CI* = [0.04, 2.24], *p* = 0.042), and Experiment (*b* = 2.60, *CI* = [0.92, 4.28], *p* = 0.003), while there was no interaction between Experiment and Context (*p* = 0.64).

To determine when the offset of the CNV and the onset of late positivity begin, we examined the crossing latency, which is the point at which the CNV waveform changes from negative to positive after 650 ms from the onset. We found significant differences in crossing latency between the two experiments, despite using the same probe duration (all *p*s < 0.001): 703 vs. 914 ms for the 400-ms target interval, 1210 vs. 1628 ms for the 1000-ms target interval, and 1860 vs. 2074 ms for the 1600-ms target interval for Experiments 1 and 2, respectively.

### Discussion

Similar to the results of Experiment 1, we found that the mean CNV amplitude increased as the target interval increased. A comparison with Experiment 1, however, revealed the CNV is not solely based on the target interval, but also depends on the period before the decision is prompted. Interestingly, we found the rate of the CNV formation and the peak amplitude of the CNV were dependent on the context. Combining the analysis of Experiments 1 and 2 revealed that the climbing rate of the CNV is a robust indicator of context modulation. Specifically, the short context resulted in a faster rate of CNV formation, meaning the CNV began earlier in the short context compared to the long context. Our results are consistent with the notion that CNV activity reflects not only the temporal accumulator of an internal timing mechanism (Macar et al. 1999) but also temporal anticipation (Elbert et al. 1991; Ng et al. 2011), particularly for the forthcoming decision-making (Kononowicz and Penney 2016).

Interestingly, the offset late positivity component was more distinct in Experiment 2 than in Experiment 1, even for the short durations such as 400 ms, when we introduced a 300 ms gap before prompting for action. This suggests that the late positive component may better reflect the decision process when the CNV is fully evolved. As seen in LPCt, the mean amplitude negatively correlated with the target interval, similar to the recent findings of Ofir and Landau (2022), who reported that the offset response amplitude decreases as the interval increases, but levels off after the interval passes the bisection point. The late offset positivity components such as LPCt, P300, or P3 have been suggested as indicators of decision-making at post-perceptual stages (Baykan and Shi 2022; Kelly and O’Connell 2013; Ofir and Landau 2022; Polich and Kok 1995). For a bisection task, the decision could be made before the stimulus offset when the interval presentation passes the bisection point, as the uncertainty of the response (‘long’) is greatly reduced for long intervals compared to short intervals. For the short intervals, the online monitoring of the passage of time and comparison to the bisection point remain active (Lindbergh and Kieffaber 2013). Thus, as suggested by Ofir and Landau (2022), the amplitude of the late positivity may reflect the distance from the decision threshold.

Most importantly, we observed contextual modulation of the late positivity LPCt component: higher amplitude but later latency for the long AF context than the short DF context. Given that LPCt amplitude negatively correlated with the target duration, a higher amplitude in the long context (AF) indicates that intervals were perceived shorter as compared to the same duration in the short context (DF), closely reflecting the behavioral results. Moreover, we did not observe any correlations between the LPCt amplitude and the JND values. Therefore, the LPCt amplitude measured in this study is likely to reflect perceived target duration, rather than task difficulty.

## General discussion

The aim of this study was to investigate the timing-related ERP components to gain a deeper understanding of neural mechanisms that underlie the influence of ensemble contexts on temporal judgments. Results showed that ensemble contexts, including stimulus spacing and sample frequency, modulated perceived time intervals, which is consistent with previous research (Penney et al. 2014; Wearden and Ferrara 1995; Zhu et al. 2021). The point of subjective equality (PSE) was biased towards the mean of the ensemble distribution, with shorter contexts leading to a lower PSE. Consequently, participants were more likely to perceive the target intervals more as “long” in shorter contexts compared to longer contexts. EEG analysis further revealed that ensemble contexts affect the climbing rate of the contingent negative variation (CNV) as well as the latency and the amplitude of the late post-offset positivity related to timing (LPCt). These components are commonly associated with expectancy and decision processes related to timing.

### The CNV

In both experiments, we observed sustained negativity, known as CNV, which emerged after the post-onset P2, peaked around 600–800 ms, and dissipated at the end of the stimulus presentation. The CNV has been considered a robust signal for temporal processing, and early studies have suggested that its evolving slope and amplitude reflect the passage of time (Macar and Besson 1985; Macar and Vitton 1982). Our results revealed that longer durations elicited prolonged sustained negativities compared to shorter durations. However, the CNV represents more than just timing. For example, when comparing brain activity between two experiments, we found that the sustained negativity elicited by the same duration was nearly 300 ms longer in Experiment 2 than in Experiment 1, primarily due to the presence of a 300-ms blank period before the cue display for response in Experiment 2. This modulation of response delay by the cue display supports the early proposition that the dissipation of the CNV may also indicate readiness for quick action (Loveless and Sanford 1974; Näätänen 1970).

Recently, Kononowicz and Penney (2016) have echoed this idea that the CNV is not solely related to timing but also influenced by more complex cognitive processes such as anticipation, expectation, and response preparation (Kononowicz et al. 2018; Kononowicz and Penney 2016; van Rijn et al. 2011). For example, Mento et al. (2013) showed that even in the absence of a motor response preparation, in a passive viewing task, CNV peaks at the point of time with the highest probability of stimulus presentation. In another study where participants were cued to respond quickly (speed trials) or accurately (accuracy trials), the CNV amplitude was more negative in speed trials than in accuracy trials (Boehm et al. 2014), suggesting that CNV amplitude may reflect changes in participants’ cautiousness towards quick decision-making. Similarly, Ng et al. (2011) demonstrated that CNV activity for the current long interval plateaued after surpassing a memorized internal criterion (around the geometric mean of sample intervals). In both experiments, our results also indicated that the mean amplitude of CNV increased with longer target intervals, leveling off around the middle intervals (Figs. 3 and 7), suggesting a close association between the CNV amplitude and the expected decision criterion.

Another significant finding is the contextual modulation of the climbing rate of CNV. In both experiments, the short context led to a faster formation of CNV compared to the long context. Since the CNV climbing rate was determined at the beginning of the presentation when the stimulus length was unknown, it reflects the general expectation of when the decision interval (the ensemble mean of the sample distribution) might occur within a given block. Thus, the rate difference between short and long contexts indicates whether the internal decision interval shifts earlier or later. Although we observed context differences in peak latency in Experiment 1 and peak amplitude in Experiment 2, as well as some numerical differences in the mean CNV amplitude, the effects were not consistently significant across both experiments.

Together, our results suggest that CNV reflects the readiness or expectation to respond to an incoming stimulus, similar to previous research (Boehm et al. 2014; Kononowicz and Penney 2016; Ng et al. 2011), and the climbing rate of initial CNV formation serves as a reliable indicator of how the decision threshold is modulated by context factors.

### The offset positivity components (P2 and LPCt)

After prompting for a response, we saw an offset positivity waveform, peaking at 200–400 ms and lasting for over 600 ms after the stimulus presentation. This offset positivity is known as P2 (Kononowicz and van Rijn 2014; Tarantino et al. 2010), P3/P3b (Ofir and Landau 2022), or LPCt (Paul et al. 2011; Wiener and Thompson 2015) depending on studies. Depending on the timing of the response cue, either immediately after the duration stimulus or after a 300-ms gap, we observed that an offset P2 (no gap) or LPCt (with a gap) were related to the temporal decision. Short intervals, relative to long intervals, elicited delayed latency for both P2 and LPCt, and higher amplitudes for LPCt.

The early findings of time-related offset P2 came from the duration comparison studies that compared a probe interval either shorter or longer than the standard interval (Kononowicz and van Rijn 2014; Tarantino et al. 2010)—shorter intervals elicited higher amplitudes and long latencies. Using the bisection task, we only found the latency similarly dependent on duration in Experiment 1. When a decision was requested immediately after the duration presentation for the short intervals (e.g., 400 and 504 ms), the P2 amplitude was likely influenced by ongoing CNV activity. The between-experiment comparison showed that when the decision response was delayed for 300 ms (Experiment 2), the late positivity was better evolved. However, we did not find any duration-related modulation in P2. Instead, the late positivity component LPCt had a strong relationship with test durations in the decision-making stage.

Late positive components, such as LPCt or P3/P3b in prior research have been measured relative to the response (Bannier et al. 2019; Wiener and Thompson 2015) or the stimulus offset (Gontier et al. 2009; Tarantino et al. 2010) with prefrontal (Gontier et al. 2008; Paul et al. 2011) or centroparietal electrode sites (Bannier et al. 2019). The late post-positivity has been linked to the involvement of post-perceptual processes (Lindbergh and Kieffaber 2013), similar to the idea that task difficulty is involved in decision processes (Gontier et al. 2009; Paul et al. 2011). For long durations, memory and decision-making processes would be already finished at the offset, whereas for short durations, these processes would still be ongoing. This means that compared to long durations, short durations resulted in higher LPCt amplitudes and longer latencies. In this study, LPCt was measured over prefrontal electrodes relative to the onset of the response cue, i.e., 300 ms after the test duration offset. The results showed that LPCt amplitude and peak latency decreased as the target interval increased and leveled off around intermediate durations, a pattern similar to a recent study (Ofir and Landau 2022), which found that the amplitude of the late positivity correlated with the distance to the decision boundary in a drift–diffusion model (DDM). According to the DDM, the uncertainty of temporal bisection depends on the distance between the accumulated time to the decision boundary—bisection threshold. Short intervals with large uncertainty elicited high LPCt amplitudes, while long intervals with less uncertainty resulted in low amplitudes. Our findings are thus consistent with this interpretation. More interestingly, LPCt was found to be context-dependent, with short contexts leading to earlier peak latencies and lower amplitudes compared to long contexts, indicating that the decision boundary was set lower for the short context and, thus, the distance to the boundary was generally shorter.

### Context-dependent modulation

Both CNV and LPCt signals have been shown to depend on contextual modulation. Climbing of CNV activity was faster, and the amplitude and latency of LPCt were lower for short contexts compared to long contexts. Previous studies have shown temporal context can impact CNV in different ways (Damsma et al. 2021; Wiener and Thompson 2015). For instance, in a reproduction task, Wiener and Thompson (2015) found that the CNV amplitude of a current trial was linearly shifted by the duration of the previous interval, with larger negative amplitudes for longer prior durations. However, this was not the case in our Experiment 2, where the short context (DF) elicited numerically higher amplitude than the long context (AF). This suggests that the CNV amplitude is more sensitive to short-term (e.g., inter-trial duration changes) rather than long-term (e.g., session-wise changes) context modulation. In contrast to the CNV amplitude, the climbing rate of CNV formation was faster for short contexts compared to long contexts in both experiments. The CNV and climbing neuronal activity are believed to have a close relationship (Pfeuty et al. 2005), and the formation of CNV indicates how the brain encodes the timing of an upcoming event. In this study, the rate of CNV reflected the expectation of the decision threshold, which was influenced by the ensemble context.

The climbing CNV activity develops early in the perceptual encoding stage, which is tied to the memory representation of the internal criterion. In contrast, the formation of LPCt occurs during the decision stage, reflecting the comparison process of the perceived duration and the internal criterion. In this study, we showed that context affects the uncertainty of the comparison by altering the PSE towards the ensemble mean. This reduces the uncertainty of bisection for the short context in general as the test duration reaches the threshold earlier in the short relative to the long context. As a result, the amplitude and latency of the LPCt decrease. It is worth noting that the context-dependence of the amplitude and latency of the LPCt has been documented in previous research. For example, Ofir and Landau (2022) found that the late positivity remains similar in both short-range (subsecond) and long-range (supra-second) bisection tasks, even though the duration considered “short” in the long range is longer than all durations in the short range.

## Conclusion

In this study, we found that ensemble context, both sample spacing and frequency, impacted the bisection task, shifting the bisection point towards the ensemble mean. Temporal context modulation was also evident in the changes in ERPs related to interval timing. In the short context, compared to the long context, the CNV climbing rate increased, and the amplitude and latency of the LPCt were reduced. Both CNV and LPCt were linked to the given test duration, but were not limited to absolute durations. Our findings, consistent with the previous studies (Baykan and Shi 2022; Boehm et al. 2014; Ofir and Landau 2022), indicate that the CNV represents an expectancy wave for upcoming decision-making, while LPCt reflects the decision-making process, both CNV and LPCt influenced by the temporal context.

## Data Availability

The data supporting the findings of this study and the code of the statistical analysis used in the manuscript are available at G-Node (https://doi.org/10.12751/g-node.7snfwg).
